# Acquisition of tumorigenic potential and enhancement of angiogenesis in pulmonary stem/progenitor cells through Oct-4 hyperexpression

**DOI:** 10.18632/oncotarget.7285

**Published:** 2016-02-09

**Authors:** Sing-Yi Gu, Choa-Chi Ho, Yung-Kang Huang, Huei-Wen Chen, Yu-Chi Wang, Chia-Yu Kuo, Shu-Chun Teng, Wen-Mei Fu, Pan-Chyr Yang, Cheng-Wen Wu, Fu-Chuo Peng, Thai-Yen Ling

**Affiliations:** ^1^ Graduate Institute of Toxicology, College of Medicine, National Taiwan University, Taipei, Taiwan; ^2^ Department of Pharmacology, College of Medicine, National Taiwan University, Taipei, Taiwan; ^3^ Department of Internal Medicine, College of Medicine, National Taiwan University, Taipei, Taiwan; ^4^ Department of Microbiology, College of Medicine, National Taiwan University, Taipei, Taiwan; ^5^ Institute of Biomedical Science, Academia Sinica, Taipei, Taiwan; ^6^ Research Center for Developmental Biology and Regenerative Medicine, National Taiwan University, Taipei, Taiwan

**Keywords:** pulmonary stem/progenitor cells, cancer initiating cells, Oct-4, angiogenesis, angiopoietins/Tie2

## Abstract

Cancer stem cells, also known as cancer initiating cells (CICs), are considered to be responsible for tumor growth and chemoresistance. Different hypotheses have been proposed to explain the origin of CICs, including mutations in adult stem/progenitor cells or the acquisition of stem-like characteristics in differentiated cells; however, studies have yielded conflicting identification for CICs and have little information for the origin to generate CICs. Part of the difficulty in identifying CICs may stem from the fact that the CICs studied have been largely derived from cancer cell lines or well-developed tumors. In previous studies, we have reported the enrichment of mouse pulmonary stem/progenitor cells (mPSCs) by using serum-free primary selection culture followed by FACS isolation using the coxsackievirus/adenovirus receptor (CAR) as the positive selection marker. Here, we demonstrated that overexpression of the pluripotent transcription factor Oct-4 is sufficient to induce CAR^+^/mPSCs transformation, which we name CAR^+^/mPSCs^Oct-4_hi^. These transformed cells possess cancer initiating and chemoresistance potential, as well as exhibiting remarkable expression of certain proangiogenic factors, including angiopoietins (ANGs) and VEGF, and enhanced angiogenic potential. Moreover, CAR^+^/mPSCs^Oct-4_hi^ actively participated in tumor blood vessel formation and triggered a novel angiogenic mechanism, the angiopoietins/Tie2 signaling pathway. These study provide critical evidence supporting the possible origin to generate CICs, and help elucidate the pathways responsible for CICs-mediated blood vessel formation.

## INTRODUCTION

Lung cancer is a leading cause of cancer-related death worldwide, and the overall 5-year survival rate remains less than 14% [1]. Increasing evidence suggests that cancer stem cells, also known as cancer initiating cells (CICs), play critical roles in tumor growth and resistance to conventional chemotherapies, and may be responsible for tumor metastasis and recurrence [2].

CICs have been identified using different *in vitro* assays and cell biomarkers, such as side population analysis, sphere formation assay, chemoresistance, aldehyde dehydrogenase (ALDH) activity, and the cell marker CD133 [3–7]. However, these *in vitro* assays alone are not enough to demonstrate that the identified cells are in fact CICs. Therefore, certain *in vivo* assays, such as limiting dilution transplantation experiments in animal models, are used to verify the results of *in vitro* assays [7, 8]. Unfortunately, studies have yielded conflicting identification of CICs in different types of cancer [2, 9]. The discrepancies in CICs identification may be due to the fact that the studied cells derived from different cancer cell lines or well-developed tumors [9, 10]. The phenotypic and functional heterogeneity of clinical tumor samples may exacerbate the difficulty in identifying CICs [10, 11].

Different hypotheses have been proposed to explain the formation of CICs, such as mutations in adult stem/progenitor cells or the acquisition of stem-like characteristics in differentiated cells; however, the sources of cells and processes involved in the development of CICs remains unclear [12, 13]. In the *K-ras^G12D^* mutation conditional mice model, the stem cells located at the bronchioalveolar duct junction were examined as potential origin for adenocarcinoma after Cre/lox mediated activation [14]. Another study has demonstrated that Oct-4, mediated by IGF-IR signaling, can form a complex with β-catenin and Sox-2 to play a crucial role in the self-renewal and oncogenic potential of CICs in lung adenocarcinomas [15]. Additionally, co-expressing Oct-4 and Nanog in A549 lung adenocarcinoma cell line can control epithelial-mesenchymal transdifferentiation, regulate tumor initiating ability, and promote metastasis behavior [16]. Moreover, a high level of Oct-4 in non-small cell lung cancer patients has been correlated with metastasis and a lower survival rate [17]. Although these studies have demonstrated that certain pluripotent genes, Oct-4, Sox-2 and Nanog, are closely associated with tumor initiating properties, the connection between aberrant pluripotent genes expression and the generation of CICs is unclear and requires further clarification.

In this study, we generated CICs in animal model to better understand the properties and characteristics of CICs, with the hope that these findings may aid cancer research to provide insight into early diagnosis and treatment of lung cancer. In previous studies, we enriched for mouse pulmonary stem/progenitor cells (mPSCs) by using serum-free primary selection culture followed by FACS isolation using the coxsackievirus/adenovirus receptor (CAR) as the positive selection marker in the culture. These CAR^+^/mPSCs exhibited stem/progenitor properties, could differentiate into type-I pneumocytes, and possessed angiogenic potential [18, 19]. We hypothesized that CAR^+^/mPSCs could be transformed via the overexpression of Oct-4 and would then develop the typical CICs phenotype and we tested type-I pneumocytes derived from CAR^+^/mPSCs as well. In the experiments described here, we examined the characteristics of the transformed cells using *in vitro* assays, including cell cycle and telomerase activity analysis, sphere forming assay, detection of CD133 expression and ALDH activity, and chemoresistance assay. In addition, *in vivo* assays, including limiting dilution transplantation and tumor metastasis assays in SCID mice, were used to further study the characteristics of the transformed cells. Since the capacity to induce angiogenesis is another trait of CICs, endothelial tube formation assay and *in ovo* chicken chorioallantoic membrane (CAM) assay were used to evaluate the angiogenic potential of the transformed cells. Our results help elucidate a possible origin and pathway for the generation of CICs, and help uncover how CICs may regulate blood vessel formation.

## RESULTS

### Trans-fection of Oct-4 for hyperexpression in CAR^+^/mPSCs

Tissue specific stem cells are small in number yet largely responsible for tissue homeostasis. In our previous studies, we successfully identified and isolated CAR^+^/mPSCs (Supplementary Figure 1A and 1B) [18, 19]. Compared with the mouse embryonic stem cell line (E14), CAR^+^/mPSCs had low expression levels of Oct-4, Sox-2 and Nanog in PCR and real-time PCR analysis (Supplementary Figure 1C). CAR^+^/mPSCs showed the potential to differentiate into type-I pneumocytes at day 7, evidenced by their flattened cellular morphology and by the presence of the type-I pneumocyte markers, T1α and AQP5 (Supplementary Figure 1D). Thus, CAR^+^/mPSCs possess pulmonary specific stem/progenitor cell properties. These cells can be identified according to CAR expression and can be efficiently isolated using FACS.

**Figure 1:**
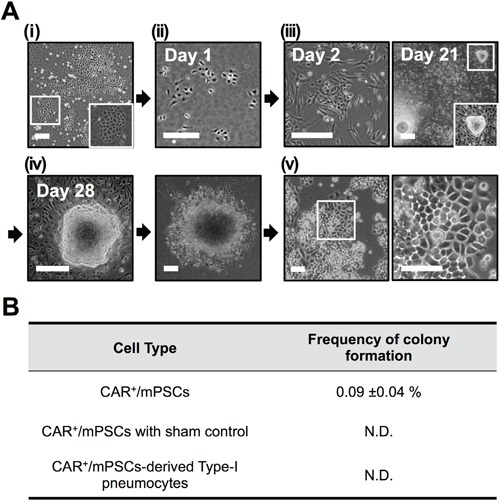
Overexpressing Oct-4 in CAR^+^/mPSCs **A.** Procedure to overexpress Oct-4 in CAR^+^/mPSCs. (i), Representative phase contrast image of primary culture. The magnified image shows the epithelial colony of mPSCs. (ii), Isolation of mPSCs according to CAR-positive expression of primary cultures using flow cytometry and subsequent transfection with retroviral vectors encoding Oct-4 cDNA. (iii), Transfected CAR^+^/mPSCs were co-cultivated with feeder cells at day 2. Cobblestone-like colonies were observed at day 21. The magnified image shows the morphology of one colony. (iv), Isolation and expansion of individual cobblestone-like colonies at day 28. (v), Colonies were established as cell clones, comprising C1, E9, and C7 clones. Representative morphology images of the C1 clone. (Scale bar, 100 μm.) **B.** Frequency of cobblestone-like colony formation. Data are presented as mean ± SD. CAR^+^/mPSCs with sham control transfection or in CAR^+^/mPSCs-derived type-I pneumocytes transfected with Oct-4 were no detectable (N.D.) colony formation.

Overexpression of Oct-4 through retrovirus transfection was performed in both CAR^+^/mPSCs and CAR^+^/mPSCs-derived type-I pneumocytes. In the experiment with CAR^+^/mPSCs transfected with Oct-4 (Figure 1A-i and 1A-ii), feeder cells were supplied at day 2 (Figure 1A-iii) and cobblestone-like colonies were first observed to form between day 18 and day 25. At day 28, the well-developed colonies exhibited phase-bright borders, and cells within the colonies had high nuclear/cytoplasmic ratios and prominent nucleoli (Figure 1A-iv). The colonies were then picked and expanded to generate cell clones (Figure 1A-v). The frequency of cobblestone-like colony formation in CAR^+^/mPSCs ranged from 0.05-0.13% (Figure 1B). Meanwhile, no cobblestone-like colonies were observed in the sham control transfection in CAR^+^/mPSCs (data not shown). Retroviral transfection of CAR^+^/mPSCs-derived type-I pneumocytes was performed at day 8 when type-I pneumocytes were well-differentiation (Supplementary Figure 2-i, 2-ii and 2-iii), and feeder cells were supplied at day 10 (Supplementary Figure 2-iv). Oct-4 transfected type-I pneumocytes had no detectable colony formation until day 42 after induction. These results indicate that overexpression of Oct-4 can induce cobblestone-like colony formation in CAR^+^/mPSCs; and that cell status, stem/progenitor stage rather than differentiated stage, appear to be critical for the induction of colonies via Oct-4 overexpression.

**Figure 2:**
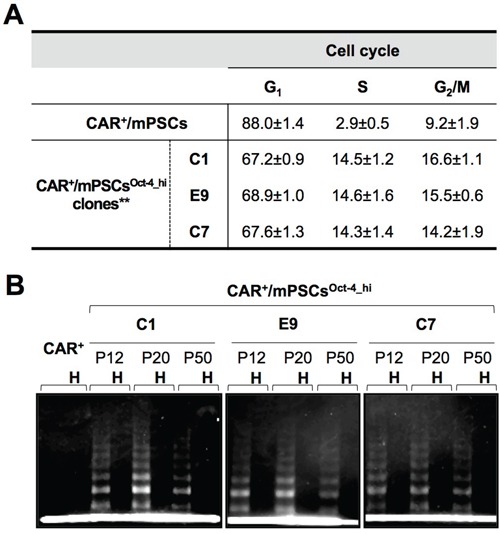
Phenotypic alterations in CAR^+^/mPSCs^Oct-4_hi^ **A.** Altered cell cycle distribution. The population for G_1_-, S-, and G_2_/M-phases of cell cycle were analyzed of CAR^+^/mPSCs and CAR^+^/mPSCs^Oct-4_hi^ C1, E9, and C7 clones. Data are presented as mean ± SD. Both the S- and G_2_/M-phase population of CAR^+^/mPSCs^Oct-4_hi^ clones were strongly increased, ** *P* < 0.01 compared with CAR^+^/mPSCs. **B.** Telomerase activity in CAR^+^/mPSCs and CAR^+^/mPSCs^Oct-4_hi^ clones. C1, E9, and C7 clones were evaluated in the 12^th^, 20^th^, and 50^th^ passages. CAR^+^ denotes CAR^+^/mPSCs. H denotes heat inactivation.

The cobblestone-like colonies were isolated and established as separate cell clones. To evaluate phenotypic alterations, cell clones from 3 independent experiments, named C1, E9, and C7, were selected for further examinations. Western blot analysis showed that Oct-4 was highly expressed in the C1, E9, and C7 clones; thus, we referred to them as CAR^+^/mPSCs^Oct-4_hi^ clones (Supplementary Figure 3-i). The Oct-4 expression levels of C1, E9, and C7 clones were similar to that of the mouse embryonic stem cell line (E14), whereas CAR^+^/mPSCs exhibited low Oct-4 expression (Supplementary Figure 3-ii). In primary cultures, CAR was specifically expressed in pulmonary stem/progenitor cells and served as the marker for CAR^+^/mPSCs isolation. In CAR^+^/mPSCs^Oct-4_hi^ clones, CAR was expressed in > 95% of cells and these cells had lost the capacity to differentiate into type-I pneumocytes (data not shown). Cell cycle analysis showed significant G_1_-, S- and G_2_/M-phase shifting between CAR^+^/mPSCs and CAR^+^/mPSCs^Oct-4_hi^ clones (Figure 2A). Additionally, the C1, E9, and C7 clones could propagate for more than 50 passages, with a doubling time of 23±1 h. While telomerase activity was detected in the 12^th^, 20^th^ and 50^th^ passages of the C1, E9, and C7 clones, it was not detected in CAR^+^/mPSCs (Figure 2B). These results demonstrate that Oct-4 hyperexpression in CAR^+^/mPSCs was sufficient to produce immortal effects, such as G_1_ cell cycle progression, proliferation potential, and telomerase activity.

### CAR^+^/mPSCs^Oct-4_hi^ exhibit tumorigenic potential

In order to evaluate the pluripotent potential of CAR^+^/mPSCs^Oct-4_hi^ clones, teratoma formation assays were performed with the C1, E9, and C7 clones. 1 × 10^6^ cells of C1, E9, or C7 clones were subcutaneously implanted in SCID mice. After 20 to 24 d, teratomas of approximately 1 cm had developed. Supplementary Figure 4 shows representative images of histopathological analysis of the C1 clone. Hematoxylin and eosin (H&E) staining showed that ectodermal, mesodermal, and endodermal lineage differentiation were absent in the tumors. Moreover, the tumors exhibited typical malignant phenotypic characteristics, such as a high cellular density; small, round immature cell proliferation; pleomorphic cells with a high nuclear/cytoplasmic ratio; and a high mitotic ratio (Supplementary Figure 4A). Using immunohistochemical staining, Oct-4 and CAR were detected in tumors (Supplementary Figure 4B). The active form of some oncogenes, including phospho-Src, phospho-β-catenin, c-myc, and cyclin D1 were also detected in the tumors (Supplementary Figure 4B). Lung adenocarcinoma diagnostic markers, such as thyroid transcription factor-1 (TTF1), Napsin A (NAPSA), cytokeratin 7 (CK7), and cytokeratin heavy molecular weight (CK-HMW) were also detected in the tumors (Supplementary Figure 4B). These data imply that CAR^+^/mPSCs acquired tumorigenic capacity through Oct-4 hyperexpression.

**Figure 3:**
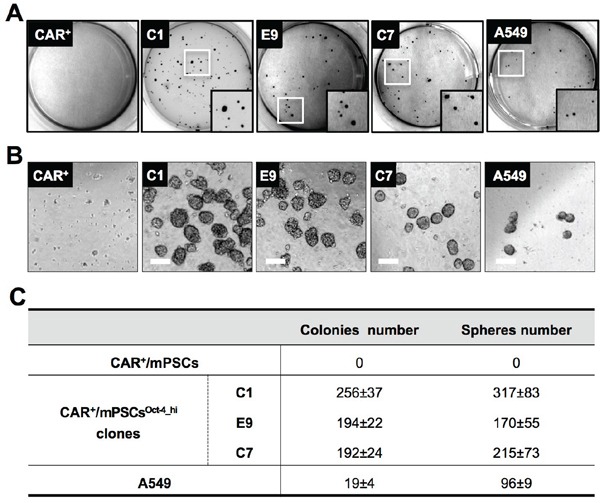
*In vitro* tumorigenic phenotype of CAR^+^/mPSCs^Oct-4_hi^ **A.** The soft agar colony formation assay. CAR^+^/mPSCs, CAR^+^/mPSCs^Oct-4_hi^ C1, E9, and C7 clones and A549 were subjected to soft agar culture. Colonies were photographed and quantified after 2 wk. **B.** Sphere formation assay. CAR^+^/mPSCs, CAR^+^/mPSCs^Oct-4_hi^ C1, E9, and C7 clones and A549 were subjected to a sphere formation assay. Secondary spheres (> 70 μm) were photographed and quantified after 10 d. (Scale bar, 100 μm.) **C.** Quantification of colonies and spheres. Data are presented as the mean ± SD.

We then quantified the tumorigenic potential of CAR^+^/mPSCs^Oct-4_hi^ clones. Anchorage-independent growth was evaluated using a soft agar colony formation assay. After 2 weeks, the C1, E9, and C7 clones had formed more significant soft agar colonies number compared to the human lung adenocarcinoma cell line A549. However, no such colonies were observed for CAR^+^/mPSCs (Figure 3A and 3C). To evaluate secondary sphere formation efficiency, C1, E9, and C7 clones were cultured under non-adhesion conditions. The secondary sphere forming efficiency of the CAR^+^/mPSCs^Oct-4_hi^ clones was significantly higher than that of A549 cells, and sphere formation was also absent in CAR^+^/mPSCs (Figure 3B and 3C).

**Figure 4:**
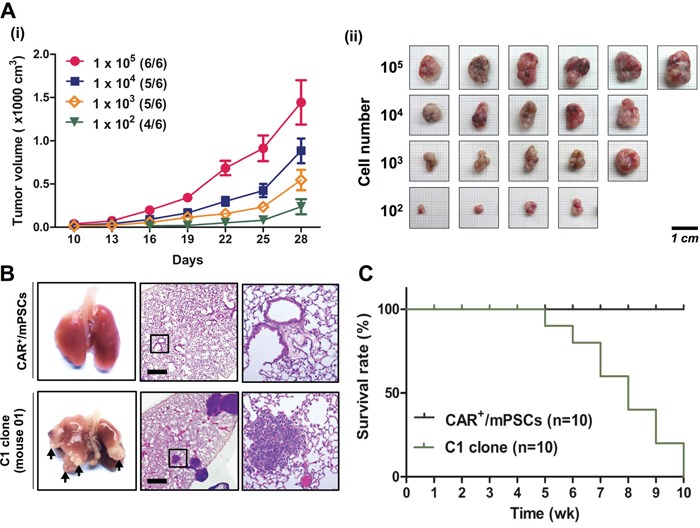
*In vivo* tumorigenic and metastatic capacities of CAR^+^/mPSCs^Oct-4_hi^ C1 clone **A.** *In vivo* xenograft tumor formation. (i), Growth curve of tumors. Different concentrations of the C1 clone (10^5^, 10^4^, 10^3^, and 10^2^ cells) were subcutaneously injected into SCID mice. Tumor diameters were measured using calipers at the indicated times after injection. Data are presented as mean ± SD. (ii), Tumors were excised, photographed, and measured at day 28 after injection. (Scale bar, 1 cm.) **B.** Metastatic tumor nodule formation. The C1 clone (3 × 10^5^ cells) was injected into the tail vein of SCID mice. CAR^+^/mPSCs were injected as a native control. Metastatic tumor nodule formation in the lung was recorded after 5 wk (indicated by arrows). H&E staining of mice injected with the C1 clone showed extensive hemorrhage and nodule formation. The magnified image of C1 clone shows nodules in lung tissue. (Scale bar, 100 μm.) **C.** Kaplan-Meier survival curves of CAR^+^/mPSCs- and C1 clone-injected mice (n = 10).

In the assays, the C1 clone exhibited the most pronounced tumorigenic behaviors, including the highest proliferation rate, highest efficiency in anchorage-independent colony formation, and secondary sphere generation; therefore, the C1 clone was selected for subsequent *in vivo* tumorigenic experiments. To determine the tumorigenicity of the C1 clone, we performed a limiting dilution transplantation experiment. Tumor formation potencies were 6/6, 5/6, and 5/6 in 10^5^, 10^4^, and 10^3^ cell concentrations of C1 clone injections, respectively. In addition, a low cell concentration of C1 clone (10^2^) was sufficient for tumor formation (4/6) at an average of 28 d after injection (Figure 4A-i). Tumor size and morphology are shown in Figure 4A-ii. In contrast, no tumor formation was observed in transplants using CAR^+^/mPSCs (10^6^ cells) despite 56 d incubation (data not shown). In order to examine metastatic potential, 3 × 10^5^ cells of the C1 clone were transplanted through the tail vein of mice and allowed to develop for 35 d. All mice injected with the C1 clone developed tumor nodules in the lung tissue (Figure 4B). H&E staining of lung tissue revealed extensive hemorrhage and nodule formation in the C1 clone transplants, while no abnormal lesions were detected after CAR^+^/mPSCs transplantation. Kaplan-Meier survival analysis was performed to determine the survival rate following C1 clone or CAR^+^/mPSCs transplantation. The mean survival of mice injected with the C1 clone was significantly lower than that of mice transplanted with CAR^+^/mPSCs (Figure 4C). These results suggest that CAR^+^/mPSCs undergo malignant transformation following Oct-4 hyperexpression, as evidenced by the *in vitro* and *in vivo* tumorigenic potential and tumor initiating capacity of the CAR^+^/mPSCs^Oct-4_hi^ clones.

### CAR^+^/mPSCs^Oct-4_hi^ exhibit lung cancer CICs traits

**Figure 5:**
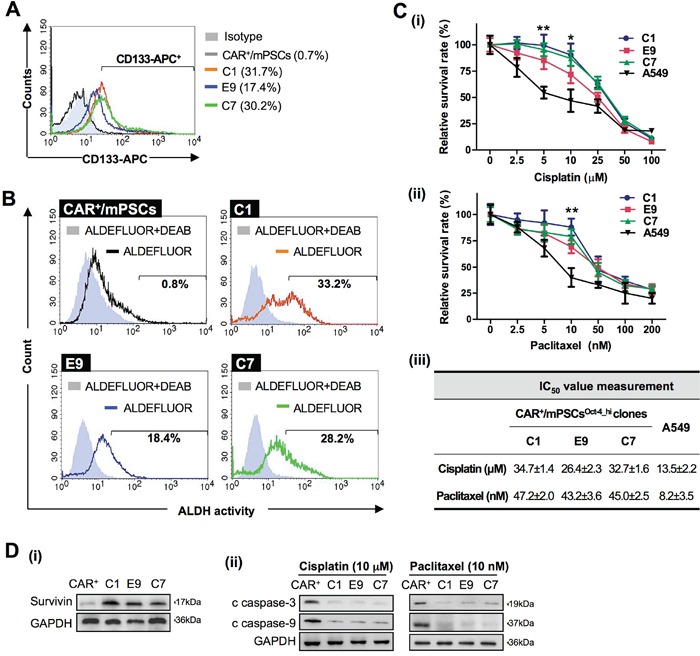
Putative CICs traits in CAR^+^/mPSCs^Oct-4_hi^ **A.** Flow cytometry analysis of CD133 expression in CAR^+^/mPSCs and CAR^+^/mPSCs^Oct-4_hi^ C1, E9, and C7 clones. Percentages indicate the CD133-positive population for each clone. **B.** Flow cytometry analysis of ALDH activity. The results of the ALDEFLUOR assay with CAR^+^/mPSCs and CAR^+^/mPSCs^Oct-4_hi^ C1, E9, and C7 clones are shown. DEAB-treated samples served as negative controls. Percentages indicate the ALDH-positive population for each clone. **C.** Cell viability of CAR^+^/mPSCs^Oct-4_hi^. CAR^+^/mPSCs^Oct-4_hi^ C1, E9, and C7 clones and A549 cells were treated with (i), cisplatin (2.5, 5, 10, 25, 50, and 100 μM) or (ii), paclitaxel (2.5, 5, 10, 50, 100, and 200 nM) for 48 h. Data are shown as the mean ± SD. * *P* < 0.05, ** *P* < 0.01 compared with A549 cells. (iii), IC_50_ of cisplatin and paclitaxel for CAR^+^/mPSCs^Oct-4_hi^ C1, E9, and C7 clones and A549 cells. Data are shown as the mean ± SD. **D.** Anti-apoptosis potential of CAR^+^/mPSCs^Oct-4_hi^ clones. (i), Survivin expression in CAR^+^/mPSCs and CAR^+^/mPSCs^Oct-4_hi^ C1, E9, and C7 clones was analyzed using Western blot. (ii), Cleaved caspase-3 (c caspase-3) and cleaved caspase-9 (c caspase-9) levels in CAR^+^/mPSCs and CAR^+^/mPSCs^Oct-4_hi^ C1, E9, and C7 clones after 10 μM cisplatin or 10 nM paclitaxel treatment.

Different biomarkers for lung CICs have been proposed, including CD133 expression, ALDH activity, and chemoresistance. We utilized these biomarkers to further investigate CICs characteristics in CAR^+^/mPSCs^Oct-4_hi^ clones. Flow cytometry analysis revealed that about 17.4-31.7% of cells were CD133^+^ among the C1, E9, and C7 clones, whereas CD133^+^ cells were nearly undetectable in CAR^+^/mPSCs (Figure 5A). ALDH activity was detected in 18.4-33.2% of the C1, E9, and C7 clones, whereas only 0.8% of CAR^+^/mPSCs exhibited ALDH activity (Figure 5B). As chemoresistance is also a critical biological feature of CICs, we evaluated the C1, E9, and C7 clones for chemoresistance to cisplatin or paclitaxel treatment. The cell viability is shown in Figure 5C. The IC_50_ values of cisplatin were 26.4-34.7 μM for C1, E9, and C7 clones, indicating that CAR^+^/mPSCs^Oct-4_hi^ clones were about 2-3 fold more resistant to cisplatin compared to the A549 cells. For paclitaxel, the IC_50_ were 43.2-47.2 nM in the C1, E9, and C7 clones, which are approximately 6 fold higher than that of the A549 cells (Figure 5C-iii). It has been well documented that the protein survivin inhibits apoptosis and plays an important role in conferring chemoresistance to CICs [20]. We found that survivin expression was significantly higher in the C1, E9, and C7 clones compared to CAR^+^/mPSCs (Figure 5D-i). The C1, E9, and C7 clones also exhibited lower levels of cleaved caspase-3 and cleaved caspase-9 under cisplatin or paclitaxel treatment compared with CAR^+^/mPSCs (Figure 5D-ii). Taken together, our results suggest that Oct-4 hyperexpression can drive the transformation of CAR^+^/mPSCs, conferring them with CICs-like properties.

### CAR^+^/mPSCs^Oct-4_hi^ participate in tumor angiogenesis

**Figure 6:**
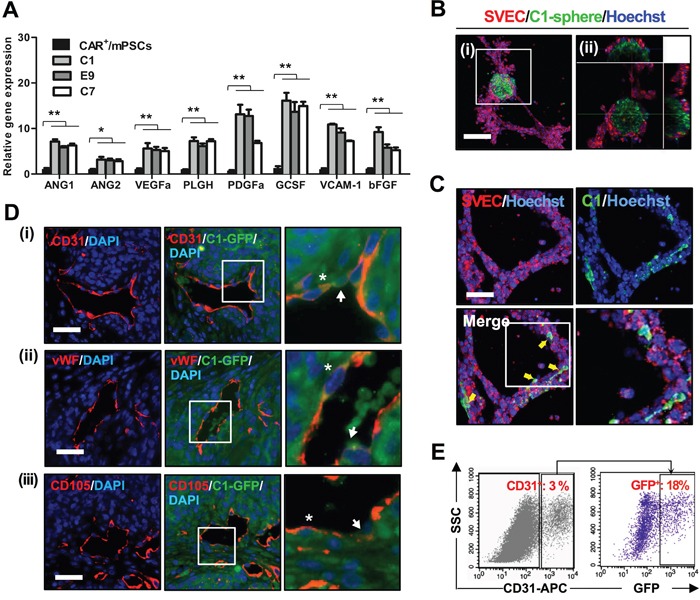
Angiogenesis potential of CAR^+^/mPSCs^Oct-4_hi^ **A.** Gene expression levels of proangiogenic factors in CAR^+^/mPSCs and CAR^+^/mPSCs^Oct-4_hi^ C1, E9, and C7 clones were analyzed using real-time PCR. Data are presented as the mean ± SD. * *P* < 0.05, ** *P* < 0.01, compared with CAR^+^/mPSCs. **B.** *In vitro* tube formation assay. SVEC4-10 cells and C1 clone-derived spheres, which stained with PKH26 and calcein-AM, respectively, were co-cultivated on Matrigel. The tube formation was recorded by fluorescence confocal microscopy for 8 h. C1 clone-derived spheres recruited SVEC4-10 cells to generate tube networks. **C.** A proportion of the C1 clone cells, indicated by arrows, were observed to integrate into the tube network. The magnified image of tube network depicts integration of the C1 clone cells. (Scale bar, 100 μm.) **D.** Immunofluorescence staining for the expression of endothelial antigens in C1-GFP clone-derived tumors. (i), CD31 was identified. (ii), vWF was identified. (iii) CD105 was identified. The magnified image shows that some GFP^+^ cells were involved in blood vessel formation (indicated by arrow), and a proportion of GFP^+^ cells also expressed CD31 (indicated by asterisk). (Scale bar, 100 μm.) **E.** CD31 expression in dissociated tumors of the C1-GFP clone was analyzed using flow cytometry. CD31^+^ sub-fraction, representing endothelial cells, constituted 3% of the whole tumor population. GFP expression was found in 18% of the CD31^+^ endothelial cell sub-fraction.

In our previous study, CAR^+^/mPSCs were shown to express the proangiogenic factors, including vascular endothelial growth factor A (VEGFa), granulocyte colony stimulating factor (GCSF), vascular cell adhesion molecule 1 (VCAM-1), and basic fibroblast growth factor (bFGF), which initiated endothelial cell tube formation [19]. Therefore, we wanted to evaluate the potential for angiogenesis in the CAR^+^/mPSCs^Oct-4_hi^ clones. We performed real-time PCR analysis and found that proangiogenic factors, including angiopoietin 1 (ANG1), angiopoietin 2 (ANG2), VEGFa, placental growth factor (PLGF), platelet-derived growth factor A (PDGFa), GCSF, VCAM-1, and bFGF, were expressed at significantly higher levels in the C1, E9, and C7 clones compared with CAR^+^/mPSCs (Figure 6A). To further confirm angiogenic potential, we used the C1, E9, and C7 clones and CAR^+^/mPSCs in a CAM assay. When implanted on CAM, the C1, E9, and C7 clones induced extensive blood vessel formation compared with CAR^+^/mPSCs implants (Supplementary Figure 5A-i). Branch point quantification revealed that implanting the C1, E9, and C7 clones significantly increased blood vessel branching compared with that of CAR^+^/mPSCs (Supplementary Figure 5A-ii). Immunohistochemical analysis and quantification of the C1, E9, and C7 clone-derived tumors revealed a significantly higher CD31-positive population than that found in A549 derived tumors (Supplementary Figure 5B-i and 5B-ii). These data suggest that Oct-4 hyperexpression may enhance the angiogenic potential in CAR^+^/mPSCs^Oct-4_hi^ clones.

**Figure 7:**
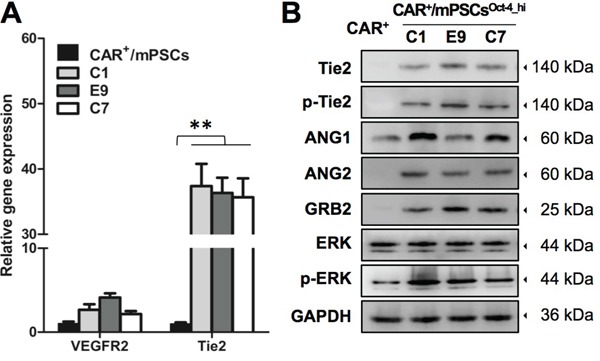
ANGs/Tie2 signaling analysis in EGM cultured CAR^+^/mPSCs^Oct-4_hi^ **A.** Real-time PCR analyzed the gene expression of angiogenesis associated receptor, including VEGFR2 and Tie2, in EGM cultured CAR^+^/mPSCs^Oct-4_hi^ C1, E9, and C7 clones and CAR^+^/mPSCs. Data are presented as the mean ± SD. ** *P* < 0.01 compared with CAR^+^/mPSCs. **B.** Western blot analyzed the ANGs/Tie2 signaling activation in EGM cultured CAR^+^/mPSCs^Oct-4_hi^ C1, E9, and C7 clones and CAR^+^/mPSCs, including Tie2, phospho-Tie2, ANG1, ANG2, GRB2, ERK and phospho-ERK expression.

To further elucidate the functional contribution of CAR^+^/mPSCs^Oct-4_hi^ in angiogenesis, we monitored tube formation of endothelial cells after incubation with the C1 clone. C1 clone-derived spheres labeled with the green fluorescent tracer, calcein-AM, were mixed with SVEC4-10 cells that had been labeled with the red fluorescent tracer, PKH26, and then were co-cultured for tube formation. C1 clone-derived spheres recruited SVEC4-10 cells and established tube network (Figure 6B). Time-lapse and 3D architecture videos using confocal microscopy are shown in Supplementary Movie-1 and -2. Some C1 clone cells were observed to integrate into the SVEC4-10 cells tube network (Figure 6C). A 3D architecture video of tube formation is shown in Supplementary Movie-3. We then cultured the C1, E9, and C7 clones in an endothelial cell growth medium (EGM; Lonza) for 7 d to examine tube formation ability; CAR^+^/mPSCs were also cultured as a control. EGM cultured C1, E9, and C7 clones exhibited tube formation ability, whereas no such capability was observed with CAR^+^/mPSCs cultured in EGM (Supplementary Figure 6-i and 6-ii). These results indicate that CAR^+^/mPSCs^Oct-4_hi^ clones not only possess angiogenic potential, but are also involved in tube formation *in vitro*. To evaluate tumor blood vessel formation potential *in vivo*, the C1 clone was transfected with GFP expression (C1-GFP clone) and then used to form tumors via subcutaneous implantation in SCID mice. Using immunofluorescence staining, the endothelial antigens, including CD31, vWF, and CD105, were detected in C1-GFP clone-derived tumors. Some blood vessels incompletely expressed the endothelial antigens, and GFP^+^ cells were directly integrated into blood vessels, exhibiting a mosaic-like pattern. Moreover, some endothelial cells simultaneously expressed endothelial antigens and were GFP-positive (Figure 6D). To confirm the presence of GFP^+^-endothelial cells, we examined dissociated tumors using flow cytometry. Similar to the results of immunofluorescence staining, 12-18% of the CD31^+^ population was also positive for GFP (Figure 6E). In order to examine the correlation between CAR^+^/mPSCs^Oct-4_hi^ clones and endothelial cells, we used real-time PCR to analyze gene expression of the angiogenesis associated receptors, VEGF receptor 2 (VEGFR2) and Tie2. The receptor, Tie2, which is specifically expressed in endothelial cells, was significantly elevated in EGM cultured C1, E9, and C7 clones compared with CAR^+^/mPSCs, while gene expression of VEGFR2 showed no significant difference (Figure 7A). Western blot analysis confirmed the involvement of the ANGs/Tie2/GRB2/ERK signaling pathway, showing that ANG1, ANG2, phospho-Tie2, GRB2, and phospho-ERK expression were significantly increased in EGM cultured C1, E9, and C7 clones relative to CAR^+^/mPSCs (Figure 7B). These data suggest that CAR^+^/mPSCs^Oct-4_hi^ actively participate in tumor angiogenesis, rather than playing a passive role as CAR^+^/mPSCs do.

## DISCUSSION

The cancer stem cell theory proposes that a small minority of tumor cells is crucial for tumor initiation and progression; these cells share common characteristics and properties with stem cells and enable tumor metastasis, recurrence, and chemoresistance [2]. Recently, in order to clarify the cancer initiating properties of the cells, cancer stem cells are also referred to as CICs [12]. Although different CICs have been identified in tumors, the origin of CICs is still unclear [9, 10]. In this article, we report that overexpressing Oct-4 at high levels in a subset of pulmonary stem/progenitor cells, CAR^+^/mPSCs, can transform the cells such that they exhibit tumorigenic potential and CICs-like properties. The transformed cells not only showed enhanced angiogenic potential, but also actively participated in tumor blood vessel formation and activated the ANGs/Tie2 signaling pathway.

Oct-4 is one of the most important transcription factors of embryonic stem cells and is also one of the four Yamanaka factors known to induce pluripotency when overexpressed in somatic cells [21]. In a study by Kim and colleagues, Oct-4 alone was sufficient to induce pluripotency in adult neural stem cells [22]. This result indicates that Oct-4 plays a critical role in maintaining cell pluripotency. However, Chiou et al. demonstrated that co-expressing Oct-4 and Nanog in A549 induced tumor initiating ability and promoted metastasis behavior of lung adenocarcinoma [16]. Beltran et al. indicated that overexpression of Oct-4 in different primary human epithelial breast cell preparations generated cell lines possessing tumor initiating and colonization capabilities, but the origin of these cells was not clearly identified [23]. Furthermore, Hochedlinger et al. reported that ectopic Oct-4 expression caused intestinal epithelial dysplasia. The dysplastic lesions showed expansion of progenitor cells and required continuous Oct-4 expression [24]. Despite these associations, the precise role of Oct-4 in cell transformation remains unclear. In our study, we found that CAR^+^/mPSCs express low levels of Oct-4, and overexpressing Oct-4 could transform CAR^+^/mPSCs such that they formed cobblestone-like colonies. In contrast, no colonies were observed when Oct-4 was also overexpressed in well-differentiated type-I pneumocytes derived from CAR^+^/mPSCs. Thus, compared with the study for the results of Oct-4 overexpression in adult neural stem cells [22], we speculated that the formation of cobblestone-like colonies via Oct-4 overexpression is dependent on cell types and differentiation status.

In this study, clones from three independent experiments were used for examinations, Western blot confirmed high expression levels of Oct-4 in the transformed cells, and thus we termed CAR^+^/mPSCs^Oct-4_hi^. In phenotypic analysis, CAR^+^/mPSCs^Oct-4_hi^ clones exhibited long-term proliferation behavior and significant shift in the cell cycle for G_1_-, S- and G_2_/M-phase indicated cell cycle progression. We also found that telomerase activity and had continuous expression in the CAR^+^/mPSCs^Oct-4_hi^ clones. Telomerase activity is considered to confer cell cycle progression and immortal growth properties [11]. Taken together, our results demonstrated that CAR^+^/mPSCs^Oct-4_hi^ clones exhibited a general phenotype of immortalized cell properties and may maintain the pluripotency. To evaluate the pluripotency of the clones, an *in vivo* teratoma formation assay, the standard tool for monitoring pluripotency, was employed [22]. Histological analysis of tissues derived from CAR^+^/mPSCs^Oct-4_hi^ clones showed no differentiation of the three germ layers. H&E staining of the tissues indicated malignant phenotypic characteristics, and immunohistological examination revealed heterogeneous phenotypes. Expression of oncogenes including phospho-Src, phospho-β-catenin, c-myc, and cyclin D1 were also detected in the tumor-like tissues, as were diagnostic markers for lung adenocarcinoma, such as TTF1, NAPSA, CK7, and CK-HMW [25, 26]. This result indicated that CAR^+^/mPSCs^Oct-4_hi^ clones induced tumor formation and derived heterogeneous tumor cell populations rather than teratoma formation.

CICs are defined by their self-renewal and pluripotency capacities, which contribute to cancer initiation, progression, metastasis, recurrence, and chemoresistance [2]. Assays that measure anchorage-independent growth and sphere formation ability are generally used to evaluate the self-renewal and differentiation capacities of CICs [4, 7]. Our results show that CAR^+^/mPSCs^Oct-4_hi^ clones possessed higher capacities for colony formation in soft agar and for sphere formation compared with the A549 cells. To demonstrate that CAR^+^/mPSCs^Oct-4_hi^ clones share certain traits with CICs, *in vivo* assays, which are regarded as the gold standard, should be performed, including limiting dilution transplantation experiments in animal models [7, 8]. Here, we used the C1 clone to demonstrate that at low concentrations (1 × 10^2^ cells), transplanted cells could generate tumors at a frequency of 4/6 within 28 d. This tumorigenicity is greater than that reported for side population of A549 cells, which at a concentration of 1 × 10^3^, gave rise to tumors at a frequency of 2/4 within 60 d [3]. Thus, CAR^+^/mPSCs^Oct-4_hi^ showed remarkable tumor initiating potential. In addition, these cells displayed the ability for metastasis and malignancy. All mice injected with the C1 clone developed tumor nodules in the lung tissue, and histopathological staining of lung tissue revealed extensive hemorrhage. In addition to the *in vitro* and *in vivo* assays, other analytic protocols used for CICs identification in lung cancer, including CD133 expression, ALDH activity, and chemoresistance assays, were used to verify the characteristics of the clones [7]. Although the mechanism underlying transformation by Oct-4 hyperexpression remains elusive, our results show that Oct-4 hyperexpression can directly transform CAR^+^/mPSCs with CICs-like properties as well as CD133 expression, higher ALDH activity, and cisplatin and paclitaxel resistance. Additionally, in our study, CAR^+^/mPSCs^Oct-4_hi^ also exhibited marked survivin expression and down regulated caspase-3 and -9 expression. It has been reported that survivin functions as a key regulator of mitosis and apoptosis inhibition and acts as an inhibitor of caspase-9 [20]. Li et al. reported that Oct-4 positively regulates survivin expression to promote cancer cell proliferation in esophageal squamous cell carcinoma [27]. We speculate that the promotion of cell proliferation and attenuated apoptosis may be critical to Oct-4 hyperexpression induced transformation and may be mediated via survivin.

It is well known that the growth, proliferation, and metastasis of cancer cells are highly dependent on angiogenesis and anti-angiogenic therapy is considered a promising strategy for cancer treatment [11]. The VEGF signaling pathway is the target of three major anti-angiogenic drugs approved for clinical use [28]. However, recent studies have revealed that some endothelial cells derived from tumor cells are resistant to anti-VEGF therapy [29–31]. Recent studies have reported that CICs may be a crucial source for key proangiogenic factors in cancers, exhibiting potent angiogenic capacities in glioma and ovarian cancers [32, 33]. In addition, Soda et al. demonstrated that the tumor initiating cells of glioblastomas presented the transdifferentiation potential for generation of tumor-derived vascular endothelial cells through hypoxia stimulation [29]. In our previous study, CAR^+^/mPSCs were demonstrated to express proangiogenic factors, such as VEGF and bFGF, and induced tube formation of endothelial cells both *in vitro* and *in vivo* [19]. In this study, we found that CAR^+^/mPSCs^Oct-4_hi^ clones expressed more abundant proangiogenic factors and induced angiogenesis in both the tube formation and the CAM assays. Surprisingly, we found that CAR^+^/mPSCs^Oct-4_hi^ clones actively participated in tube formation, creating a mosaic-like pattern, in which the C1 clone cells integrated into the tube network of SVEC4-10 cells. In an early report, Chang and colleagues showed that both endothelial cells and tumor cells formed the luminal surface to presence of “mosaic” vessels [34]. Tomaso et al. demonstrated that there was mosaic structure for endothelium of vessels in human colon carcinoma xenografts in mice [35]. In animal model studies, C1 clone derived GFP expressing cells could also participate in forming the tumor blood vessels in C1-GFP clone derived tumors. Flow cytometry analysis of C1-GFP clone derived tumors revealed that 12-18% of CD31^+^ endothelial cells also expressed GFP, indicating that a population of CD31^+^ cells was derived from the C1-GFP clone. Collectively, the results indicated that CAR^+^/mPSCs^Oct-4_hi^ exhibited remarkable angiogenic potentials and actively participated in tumor angiogenesis. In order to speculate the mechanism for CAR^+^/mPSCs^Oct-4_hi^ clones to participate in angiogenesis, our study found that CAR^+^/mPSCs^Oct-4_hi^ effectively switched on a novel angiogenic mechanism, the ANGs/Tie2 signaling pathway. ANGs are the ligand for endothelium-specific receptor tyrosine kinase Tie2 [36]. In cancer research, ANGs/Tie2 signaling plays critical roles in vascular development and maturation, which are crucial for tumor angiogenesis [36]. The underlying mechanism of ANGs/Tie2 activation in CAR^+^/mPSCs^Oct-4_hi^ clones warrants further exploration. The tumor vasculature derived from cancer cells may be regulated differently than regular vasculature, offering potential new therapeutic targets.

The hallmarks of cancer cells collectively promote survival and proliferation in foreign environments and suppress oncogenic stress, including self-sufficiency in growth signals, insensitivity to anti-growth signals, evasion of apoptosis, sustained angiogenesis, tissue invasion and metastasis, and unlimited replicative potential [11]. In this study, we demonstrated that Oct-4 hyperexpression is sufficient for CAR^+^/mPSCs to gain the hallmarks of cancer cells and acquisition of CICs-like properties. CAR^+^/mPSCs^Oct-4_hi^ clones may be valuable cell models for further studies on the properties of CICs. Moreover, the easy manipulation of CAR^+^/mPSCs^Oct-4_hi^ clones may enable large-scale cultures for the development of new therapeutic approaches targeting CICs. Although Oct-4 hyperexpression induced transformation of CAR^+^/mPSCs has been well documented, the identification of naturally occurring environmental factors that can induce Oct-4 expression warrants additional research. Recent studies have reported that a hypoxic microenvironment induced Oct-4 expression through HIF2α activation and deregulation [37]. Notably, CAR^+^/mPSCs^Oct-4_hi^ actively participated in tumor angiogenesis and activated the ANGs/Tie2 signaling pathway. These findings provide novel insight regarding the origin of CICs and offer new strategies for anti-angiogenic therapy in lung cancer.

## MATERIALS AND METHODS

### Cell culture

Human lung adenocarcinoma cell line A549, mouse axillary lymph node/vascular epithelial cell line SVEC4-10 and human embryonic kidney cell line (HEK) 293T were obtained from the Bioresource Collection and Research Center of Taiwan. A549, SVEC4-10 and HEK293T cells were maintained in Dulbecco's modified Eagle medium (DMEM, Sigma-Aldrich) with 10% FBS at 37°C in humidified incubator with 5% CO_2_.

### Oct-4 transfection

CAR^+^/mPSCs were isolated from primary cultures according to CAR-positive expression by FACS as described previously [18, 19]. Detailed methods are described in Supplementary Methods. CAR^+^/mPSCs and CAR^+^/mPSCs-derived type-I pneumocytes were transfected with retroviral vectors encoding Oct-4. Briefly, the retroviral vector plasmid pMXs-mOct-4 (Addgene) and packaging plasmids (pCMV-gag-pol-PA and pCMV-VSVg) were introduced into HEK293T cells using GeneJuice transfection reagent (Novagen). After 48 h, viral supernatants were passed through a 0.45 μm filter and supplemented with 10 μg/mL polybrene. CAR^+^/mPSCs and derived type-I pneumocytes were seeded at 1 × 10^4^ cells per 35 mm dish and incubated in the viral supernatants for 16 h. Transfected cells were cultivated in mES/MCDB201 (1:1) medium and supplied with mitomycin C inactivated MEF cells (feeder cells). Cobblestone-like colonies formed between day 18 and day 25. At day 28, colonies were manually isolated and further expanded on Matrigel (Becton Dickinson Biosciences) supplement in mES/MCDB201 (1:1) medium to establish the C1, E9, and C7 cell clones. For C1-GFP clone generation, C1 clone was transfected with retroviral vectors encoding GFP. Briefly, the retroviral vector plasmid pMXs-puro GFP (Addgene) and packaging plasmids (pCMV-gag-pol-PA and pCMV-VSVg) were introduced into HEK293T cells using GeneJuice transfection reagent (Novagen). After 48 h, viral supernatants were passed through a 0.45 μm filter and supplemented with 10 μg/mL polybrene. C1 clone were seeded at 1 × 10^4^ cells per 35 mm dish and incubated in the viral supernatants for 16 h. Puromycin (2.5 μg/mL) was add to the medium, after 5 d, GFP-positive colonies were determined for expansion, referred to as C1-GFP clone.

### RNA extraction, reverse transcription PCR, and real-time PCR

Total RNA was extracted using TRIzol (Invitrogen). For cDNA synthesis, M-MLV RT (Promega) was used according to the manufacturer's instructions. Reverse transcription PCR was performed using Taq polymerase (Invitrogen) according to the manufacture's protocol. Real-time PCR was performed using the 7900 HT real-time PCR instrument (Applied Biosystems). Primer sequences are listed in Supplementary Table S1. Glyceraldehyde 3-phosphate dehydrogenase (GAPDH) expression was used for normalization.

### Western blot analysis

Cell lysates were extracted in RIPA buffer (Pierce) and quantified by a BCA protein assay kit (Pierce) according to the manufacturer's protocol. Equal amounts (30 μg) of total protein were separated using sodium dodecyl sulfate polyacrylamide gel electrophoresis and blotted onto activated polyvinylidene difluoride membranes (Millipore). After blocking with 5% fat-free milk, the membranes were incubated with primary antibodies, as listed in Supplementary Table S2. The blots were then incubated with secondary antibody conjugated with horseradish peroxidase and immunoreacted bands were detected by enhanced chemiluminescence detection (Millipore).

### Flow cytometry analysis

In CD133 expression analysis, cells were dissociated into single cells, washed, and suspended in PBS. Cells were labeled with allophycocyanin (APC)-conjugated anti-mouse CD133 (BioLegend), and then analyzed using the FACS caliber instrument. In cell cycle distribution analysis, cells were cultivated in 6-well plates. After incubating for 24 h, cells were collected, washed with PBS, and fixed in 70% ethanol at −20°C overnight. Subsequently, the cells were washed once with PBS and re-suspended in PBS containing 200 μg/mL RNase A and 50 μg/mL propidium iodide. FACS caliber instrument analyzed the cell cycle distribution. CD31 and GFP expression in tumors, tissue dissociation kit (Miltenyi Biotec) was used to dissociate tumors into cell suspension, according to the manufacturer's protocol. Cell suspension was stained with APC conjugated anti-mouse CD31 (BioLegend) and subsequently analyzed using the FACS-caliber instrument.

### ALDH activity assay

The ALDH activity of cells was detected using the ALDEFLUOR assay kit (StemCell Technologies) according to the manufacturer's protocol. The cells were suspended in an ALDEFLUOR assay buffer containing BODIPY-aminoacetaldehyde (BAAA) and incubated for 60 min at 37°C. The cells were treated with an ALDH inhibitor, diethylaminobenzaldehyde (DEAB), as a negative control. Propidium iodide staining identified nonviable cells. The FACS-caliber instrument was used to analyze the ALDH activity of cells in a green fluorescence channel (520-540 nm).

### Telomerase repeat amplification assay

Telomerase activity was measured using the telomerase repeat amplification (TRAP) assay. Cells were homogenized in a TRAP lysis buffer. Protein (20 μg) was used in the telomerase reaction, along with 50 μL of a TRAP reaction buffer containing 20 mM Tris-HCl (pH 8.3), 1.5 mM MgCl_2_, 63 mM KCl, 0.05% Tween-20, 1 mM EGTA, 50 μM deoxynucleotide triphosphate (Pharmacia), 0.1 μg each of labeled TS, ACX, and U2 primers, 5 × 10^−3^ attomoles of an internal control primer (TSU2), 2 units of Taq DNA polymerase (Invitrogen), and 2 μL of CHAPS extract. After incubating at 3°C for 30 min, the telomerase-extended products were amplified through PCR under the following conditions: 30 cycles with each cycle comprising incubations at 94°C for 30 s, 60°C for 30 s, and 72°C for 45 s. The reaction mixture was heated to 94°C for 5 min to inactivate telomerase. Amplified products were resolved on a 12% polyacrylamide gel electrophoresis, stained with ethidium bromide and viewed under UV light.

### Soft agar colony formation assay

A soft agar colony formation assay was performed by seeding 3 × 10^3^ cells in 35 mm tissue culture dishes containing a layer of 0.35% low-melting agarose/ES/MCDB-201 over a layer of 0.5% low-melting agarose/ES/MCDB-201. Additional complete media was added every 2 d. After 2 wk, colonies were fixed with 0.05% crystal violet and methanol and colony formation was photographed and quantified using light microscopy.

### Sphere formation assay

Cells were seeded in a 24 well ultra low-attachment plate (Corning) at a density of 1,000 cells per well and grown in serum-free DMEM, supplemented with 2% B27 (Invitrogen), 20 ng/mL EGF, and 20 ng/mL bFGF (Invitrogen). After cultivation for 14 d, primary spheres were harvested using centrifugation, dissociated with trypsin, and re-suspended in this medium. The secondary spheres (> 70 μm) were photographed and quantified after 10 d.

### Xenograft tumor assay

All animal experiments were reviewed and approved by the Institutional Animal Care and Use Committee of National Taiwan University College of Medicine. For teratoma formation assay, 1 × 10^6^ cells of CAR^+^/mPSCs^Oct-4_hi^ C1, E9, and C7 clones were subcutaneously injected into 8-week-old male severe combined immunodeficiency (SCID) mice. For limiting dilution transplantation experiment, the C1 clone (10^5^, 10^4^, 10^3^ and 10^2^ cells) or CAR^+^/mPSCs (10^6^ cells) were subcutaneously injected into SCID mice. For C1-GFP clone and A549 cells derived tumor experiments, C1-GFP clone (1 × 10^5^ cells) or A549 (1 × 10^6^ cells) were subcutaneously injected into SCID mice. Tumor dimensions were measured using calipers once every 3 d, and volumes (cm^3^) were calculated according to the standard formula: length × width^2^/2. At the end of the experiment, the tumors were surgically excised and photographed. In metastasis assay, 3 × 10^5^ cells of C1 clone and CAR^+^/mPSCs were injected into the lateral tail vein of SCID mice. Lung metastatic nodules were evaluated at week 5 by necropsy and histological examination. Kaplan–Meier analysis was used for comparing the survival rates of mice injected with C1 clone and those injected with CAR^+^/mPSCs.

### Immunohistochemistry and immunofluorescence staining

Tumors were fixed in formalin and subsequently dehydrated, paraffin embedded, and sectioned. Tumor sections were subjected to antigen retrieval with microwave irradiation in a citrate buffer (10 mM, pH 6.0). The sections were incubated at 4°C with primary antibody overnight. For immunohistochemical staining, the sections were incubated with corresponding HRP-coupling secondary antibodies at room temperature for 1 h, and visualized using 0.05% 3,3′-diaminobenzidine (DAB), and the nuclei were counterstained with hematoxylin. For immunofluorescence staining, corresponding fluorescence coupling with a secondary antibody was performed at room temperature for 1 h. The nuclei were counterstained with DAPI. Negative controls were prepared using identical conditions, and control IgG was used as a substitute for the primary antibody. Antibodies are listed in Supplementary Table S2. Sections were examined using the Nikon Eclipse 800. Immunohistochemical staining sections were quantified using TissueFax (TissueGnostics GmbH) scanning, and the percentage of immune-positive population were analyzed with HistoQuest software (TissueGnostics GmbH).

### Cell viability assay

Cells were seeded at 3 × 10^3^ cells per well in 96-well plates and incubated for 18 h. Cells were treated with cisplatin (Sigma-Aldrich) or paclitaxel (Sigma-Aldrich) at various concentrations. After 48 h, WST-1 assay (Roche) was performed to determine cell viability according to the manufacturer's instructions. Cell viability was expressed as a percentage of the non-treated group, and the IC_50_ values were determined.

### Chick chorioallantoic membrane (CAM) assay

Fertilized chicken eggs were incubated at 37°C in an atmosphere of 80% humidity. At day 8 of development, 1 × 10^6^ cells were loaded onto a membrane and implanted on the top of the growing CAM. At day 11, CAM was fixed with 4% paraformaldehyde, and photographed using a stereomicroscope and digital camera. Branching points were quantified using NIH Image J software with the angiogenesis plugin.

### *In vitro* CAR^+^/mPSCs^Oct-4_hi^ tube formation assay

Cells were cultivated in an endothelial cell growth medium (EGM) (Lonza) for 7 d. Cells were collected and suspended in DMEM supplemented with 2% FBS and seeded on Matrigel. After 8 h, cells were stained with calcein-AM (Invitrogen), and images were obtained using a fluorescence microscope (Zeiss).

### CAR^+^/mPSCs^Oct-4_hi^ C1 clone and SVEC4-10 co-culture for tube formation

Matrigel was plated on 35 mm Ibidi μ dishes. C1 clone derived spheres labeled with the green fluorescent tracer, calcein-AM, were mixed with SVEC4-10 cells that had been stained with a red fluorescent cell tracer dye, PHK26 (Sigma-Aldrich). Nuclei were counterstained with Hoechst33342. The cellular mixture was seeded onto Matrigel plated dishes in DMEM containing 2% FBS and 5% Matrigel. Tube formation was recorded using time-lapse immunofluorescence confocal microscopy.

### Statistical analysis

Quantitative data from at least three independent experiments are expressed as means ± standard deviation (SD). Student's *t*-tests were used to compare the differences between groups. Survival curves were obtained using the Kaplan-Meier analysis. *P* < 0.05 is considered statistically significant.

## SUPPLEMENTARY DATA FIGURES, MOVIES AND TABLES








