# Supplementary material for: Acquisition of tumorigenic potential and enhancement of angiogenesis in pulmonary stem/progenitor cells through Oct-4 hyperexpression
Source: Oncotarget. 2016 Feb 9;7(12):13917–31. doi: 10.18632/oncotarget.7285 (PMC4924688; doi:10.18632/oncotarget.7285)
Supplement: Supplementary file 1 [file oncotarget-07-13917-s001.pdf]

SUPPLEMENTARY DATA

Serum-free primary selection culture of mouse pulmonary stem/progenitor cells

Neonatal ICR mice (postnatal between 1 to 3 days) were sacrificed by cervical dislocation. The lung tissue was separated and collected in pre-chilled Hank's buffer with penicillin (100 units/mL) and streptomycin (100 µg/mL). Lung tissues were cut into small pieces of 1 to 2 mm in diameter in digested medium containing 0.1% protease type-XIV (Sigma-Aldrich) and 1 ng/mL DNase-I (Sigma-Aldrich) in Minimum Essential Medium Eagle (MEM) medium at 4°C overnight. Afterward, 10% FBS/MEM medium was added to neutralize the protease/DNase-I and tissues suspension were gently pipetted with 10-mL pipettes several times. Tissue debris was filtered through a 100 µm nylon cell strainer. The cells were washed and resuspended in MCDB-201 medium (Sigma-Aldrich) supplemented with insulin/transferrin/selenium (ITS) (Invitrogen). These cells were cultivated at a density of 3×10^5 cells/mL in collagen-I (Becton Dickinson Biosciences) coated cell culture dishes. After 1 day of incubation, the cells were refreshed on MCDB-201 medium supplemented with ITS and recombinant 1 ng/mL epidermal growth factors (Invitrogen). Pulmonary epithelial colonies formed in the culture when cells were confluent at day 10 to 14. These primary cells were applied to CAR-positive mPSCs isolation using FACS.

CAR$^+$/mPSCs isolation

Cell suspensions obtained from the primary cultures were analyzed for CAR-positive cells using a FACS caliber instrument (Becton Dickinson Biosciences). Briefly, 1×10^6 cells were incubated with goat polyclonal anti-CAR antibody (R&D Systems) at 4°C for 1 h. After washing, cells were incubated with Alexa488-coupled donkey anti-goat IgG (Jackson ImmunoResearch) at 4°C for 1 h. Cell fluorescence was evaluated using an FACSAria™ cell sorter (Becton Dickinson Biosciences), and data were analyzed using CellQuest™ (Becton Dickinson Biosciences). Cells were purified to > 90% according to CAR-positive expression, and referred to as CAR$^+$/mPSCs. CAR$^+$/mPSCs were centrifuged using low speed centrifugation (1100 rpm for 5 min) and re-suspended for later use, including Oct-4 transfection and cell differentiation experiments.

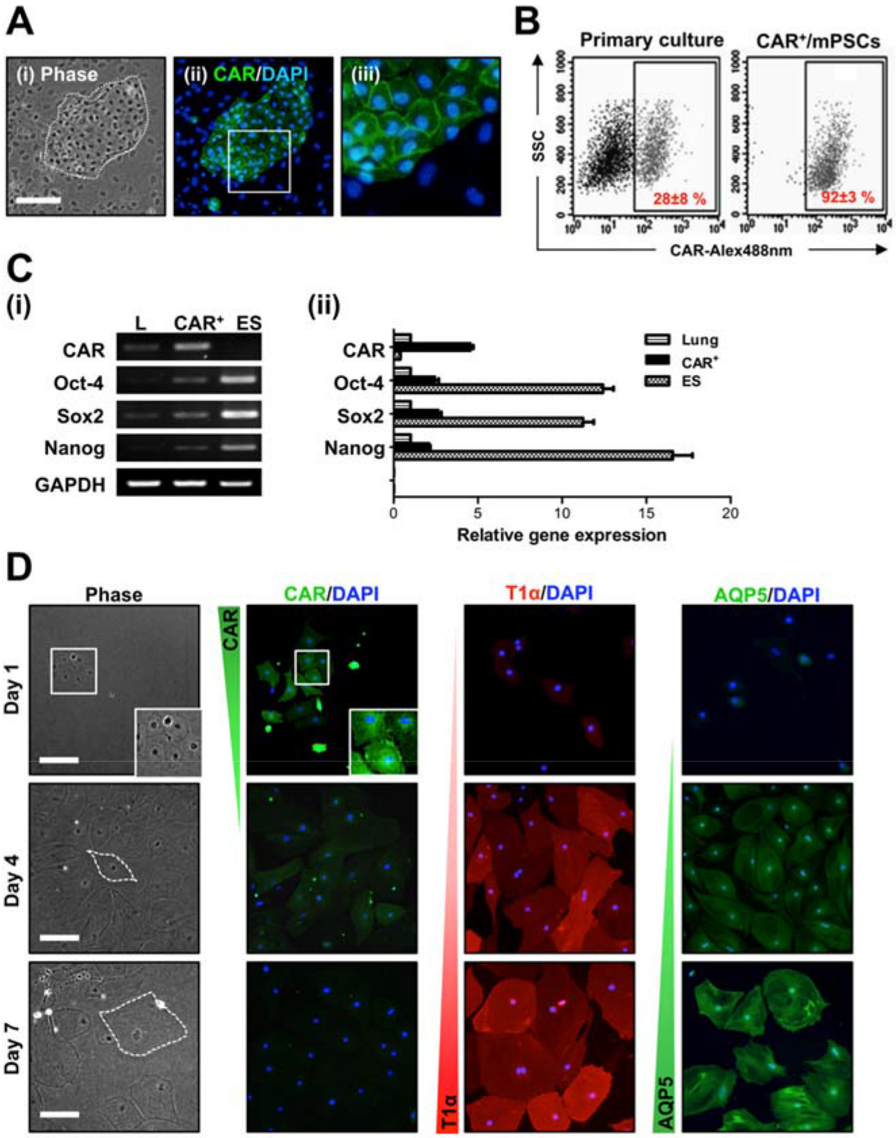

Supplementary Figure 1: CAR$^+$/mPSCs culture, isolation and differentiation. A. Immunofluorescence staining of CAR in the epithelial colonies of primary cultures. (i), The epithelial colony is denoted by the white dotted line in the phase contrast image. (ii), Immunofluorescence images showing CAR expressed at cell-cell junctions of the epithelial colony. (iii), Magnified image of the boxed area in panel A-ii. (Scale bar, 100 µm.) **B.** The CAR-positive population of the primary culture was identified and isolated using FACS, referred to as CAR$^+$/mPSCs. **C.** Gene expression profiles of CAR$^+$/mPSCs were analyzed using PCR and real-time PCR. Gene expression of CAR, Oct-4, Sox-2, and Nanog were evaluated. L, mouse lung tissue; CAR$^+$, CAR$^+$/mPSCs; ES, mouse embryonic stem cell line (E14). Data are expressed as the mean ± SD. **D.** CAR$^+$/mPSCs differentiation. CAR$^+$/mPSCs differentiated into type-I pneumocytes for 7 d after isolation. At day 1, the magnified image shows the isolated cells in the boxed area. White dashed lines indicate the phase contrast images of the differentiated cells at day 4 and 7. The expression of CAR and type-I pneumocyte markers, T1α and AQP5, were evaluated using immunofluorescence staining. CAR expression was detected at day 1 and the magnified image of the boxed area shows CAR expression at the cell-cell junctions of isolated cells. At day 4 and day 7, CAR expression was absent. T1α and AQP5 expression were detected at day 4 and day 7. (Scale bar, 100 µm.)

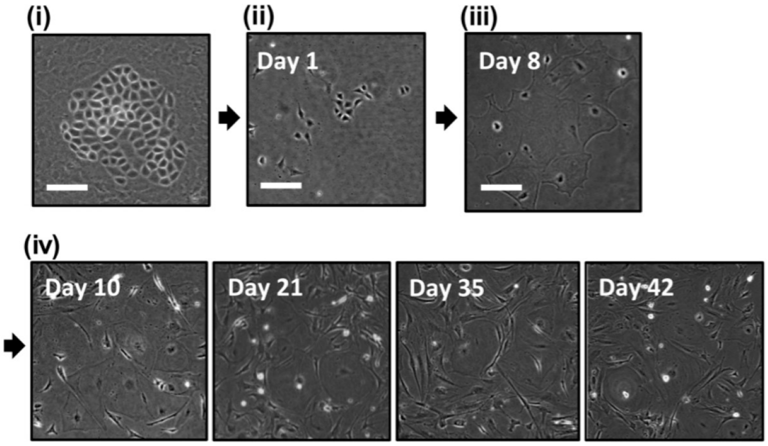

Supplementary Figure 2: Overexpression of Oct-4 in type-I pneumocytes. Time course of the Oct-4 overexpression procedure in CAR^+/mPSCs-derived type-I pneumocytes. (i), Representative phase contrast image of primary cultures showing epithelial colony. (ii), CAR^+/mPSCs underwent differentiation into type-I pneumocytes for day 7. (iii), At day 8, type-I pneumocytes were transfected with retroviral vectors encoding Oct-4 cDNA. (iv), Transfected cells proliferated upon addition of a feeder cell supplement at day 10, 21, 35, and 42. (Scale bar, 100 μm).

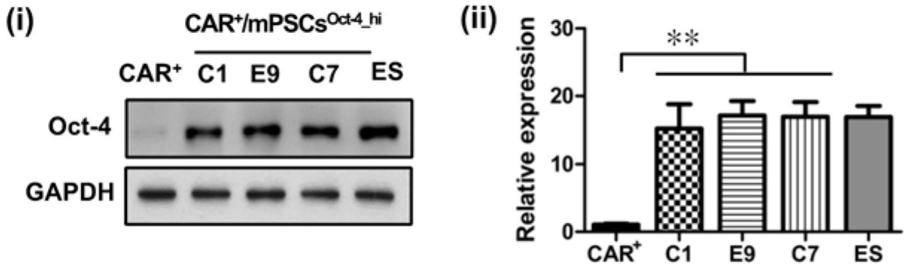

Supplementary Figure 3: Oct-4 hyperexpression in CAR^{+}/mPSCs$^{Oct\text{-}4_hi}$. (i), Oct-4 expression in CAR^{+}/mPSCs and CAR^{+}/mPSCs$^{Oct\text{-}4_hi}$ C1, E9, and C7 clones were analyzed using Western blot. ES denotes mouse embryonic stem cell line (E14). (ii), Quantification of Oct-4 expression. Data are presented as the mean ± SD. ** $P < 0.01$ compared with CAR^{+}/mPSCs.

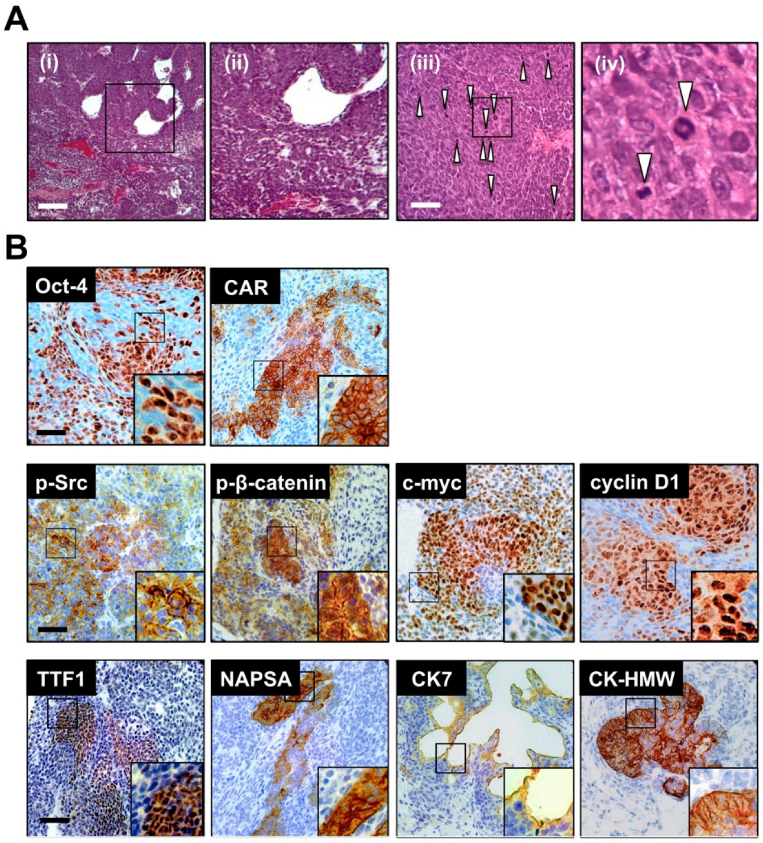

Supplementary Figure 4: Immunohistochemical examination of C1 clone-derived tumors. **A.** Representative H&E stained images of C1 clone-derived tumors. (i), Cells with a high nuclear/cytoplasmic ratio are shown. (ii), Magnified image of the boxed area in plane A-i. (iii), Tumor cells with a high mitotic rate are indicated with arrow heads. (iv), Magnified image of the boxed area in plane A-iii. (Scale bar, 100 μm.) **B.** Immunohistochemical examination for the expression of Oct-4 and CAR, and tumor associated molecules, including phospho-Src, phospho-β-catenin, c-myc, cyclin D1, TTF1, NAPSA, CK7, and CK-HMW, in C1 clone-derived tumors. Insets are magnified images of boxed areas. (Scale bar, 100 μm).

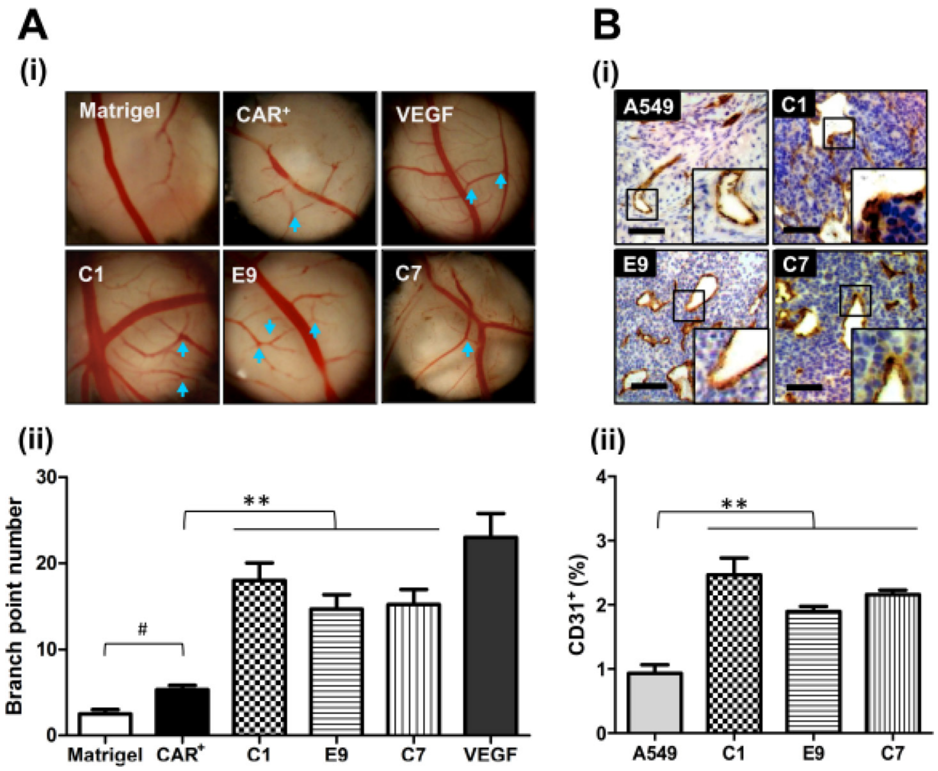

Supplementary Figure 5: Angiogenic potential of CAR^{+}/mPSCs$^{Oct\text{-}4_hi}$. A. CAM assay of CAR^{+}/mPSCs and CAR^{+}/mPSCs$^{Oct\text{-}4_hi}$ C1, E9, and C7 clones. (i), Representative photomicrographs of cell transplantation after 72 h. Arrows indicate the branching points of blood vessels. (ii), Angiogenic potential was determined by counting the branch points. Matrigel alone was used to determine the background level and VEGF (10 ng) was used as a positive control. Data are expressed as the mean ± SD. # $P < 0.05$ compared with Matrigel alone. ** $P < 0.01$ compared with CAR^{+}/mPSCs. **B.** Immunohistochemical staining of CD31 expression in CAR^{+}/mPSCs$^{Oct\text{-}4_hi}$ C1, E9, and C7 clones and A549 derived tumors. (i), Representative images of each tumor are shown. (Scale bar, 100 μm.) (ii), Quantification of CD31 expression in the tumors by TissueGnostics scanning and HistoQuest software analysis. Data are expressed as the mean ± SD. ** $P < 0.01$ compared with A549 tumors.

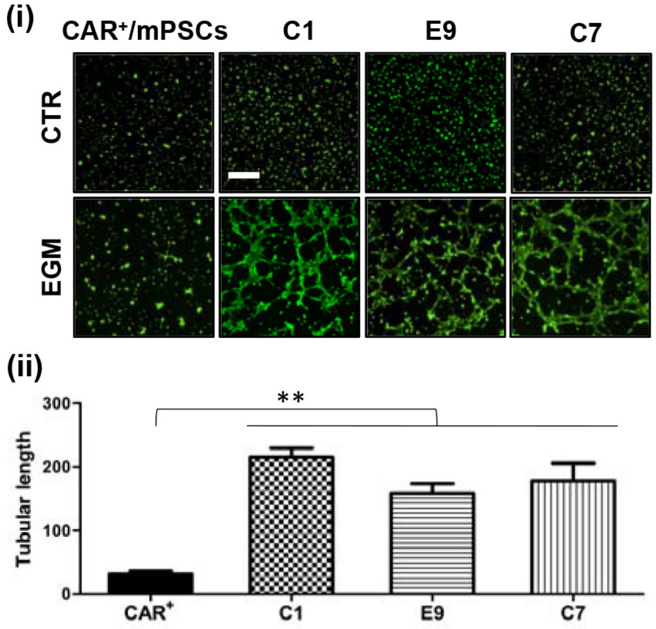

Supplementary Figure 6: Tube formation assay for CAR⁺/mPSCs$^{Oct\text{-}4_hi}$. (i), After incubation in EGM for 7 d, tube formation was detected in CAR$^+$/mPSCs$^{Oct\text{-}4_hi}$ C1, E9, and C7 clones. No tube formation was observed with EGM cultured CAR$^+$/mPSCs. The tube network was staining using calcein-AM and recorded by fluorescence microscopy for 8 h. (ii), Tube formation capacity was determined by quantifying the tubular length. Data are expressed as the mean ± SD. ** $P < 0.01$ compared with CAR$^+$/mPSCs. (Scale bar, 100 μm).

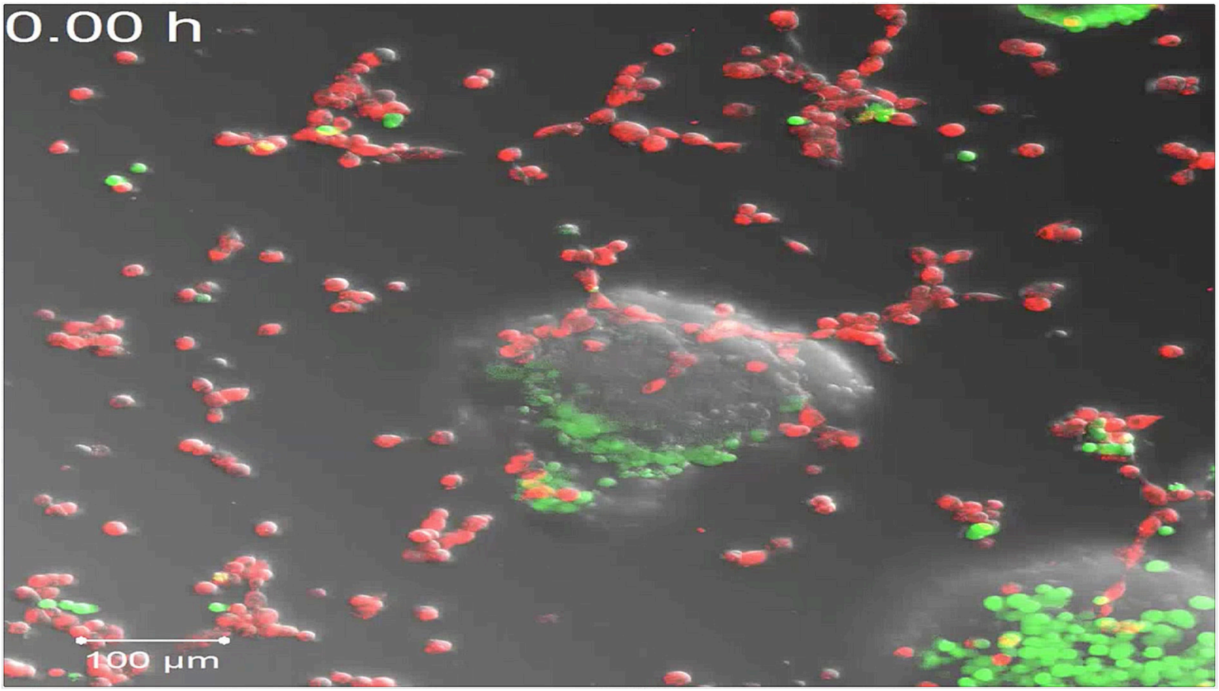

Supplementary Movie-1: Time-lapse photography of tube formation *in vitro*. SVEC4-10 cells and C1 clone-derived spheres, which stained with PKH26 and calcein-AM, respectively, were co-cultivated on Matrigel to evaluate the angiogenic potentials of C1 clone. The process of tube formation was recorded by time-lapse laser-scanning confocal fluorescence microscopy (LSM780, Carl Zeiss) and analyzed through ZEN software (Carl Zeiss). C1 clone derived sphere could recruit SVEC4-10 cells to generate tube networks in 8 h. (Scale bar, 100 µm).

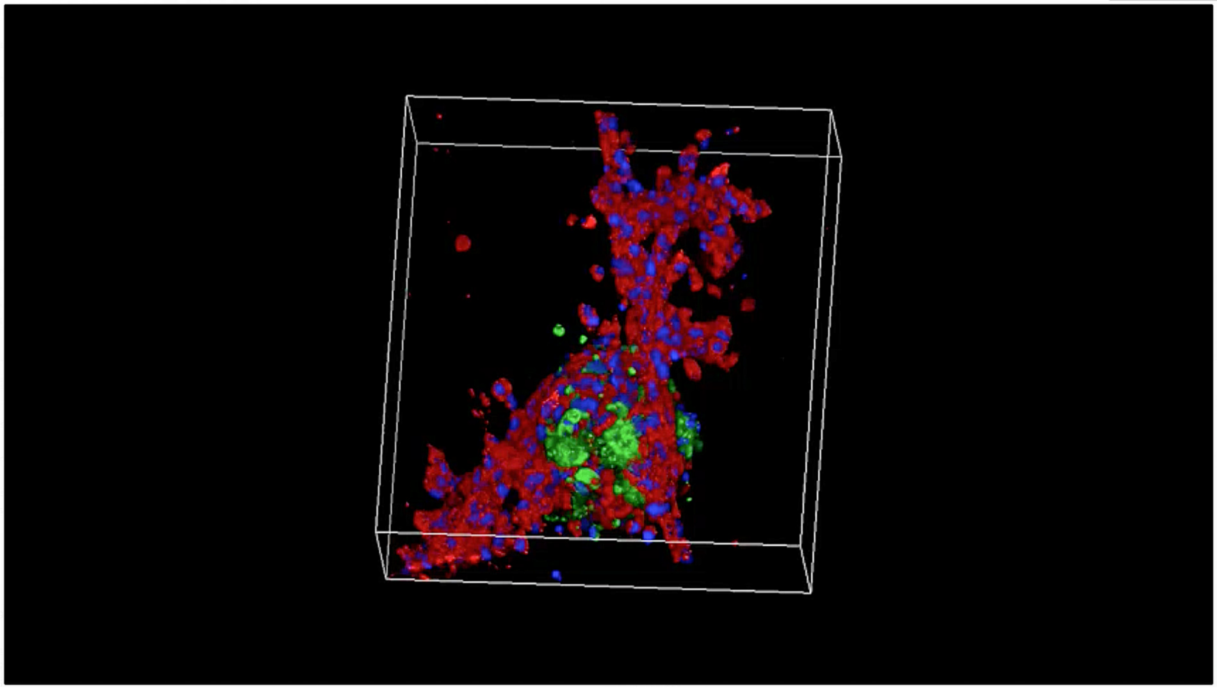

Supplementary Movie-2: The 3D architecture image of tube networks for the co-culture of PKH26-labeled SVEC4-10 cells and calcein-AM-labeled C1 clone-derived spheres.

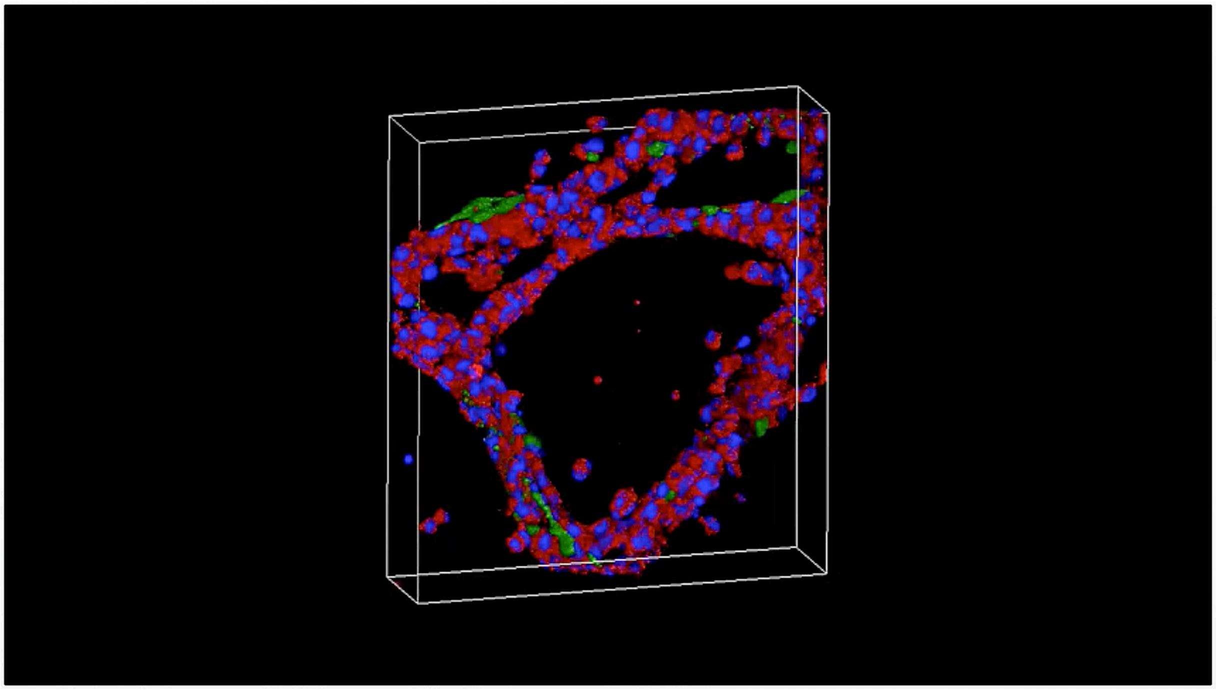

Supplementary Movie-3: 3D architecture image to show the integration of C1 clone cells into the tube networks generated by co-culture of SVEC4-10 cells and C1 clone-derived spheres.

Supplementary Table 1: RT-PCR and Real-time primer sequence

Gene	Accession	Forward Primers (5'→3')	Reverse Primers (5'→3')	AT (°C)	Product Size (bp)
**RT-PCR**					
CAR	NM_009988	CGATGTCAAGTCTGGCGA	GAACCGTGCAGCTGTATG	57	356
Oct-4	NM_013633	ATGGCTGGACACCTGGCTTC	CCAGGTTCTCTTGTCTACCTC	62	1121
Sox2	NM_011443	TAGAGCTAGACTCCGGGCGATGA	TTGCCTTAAACAAGACCA	62	297
Nanog	NM_028016	AAAGGATGAAGTGCAAGCGGTGG	CTGGCTTTGCCCTGACTTTAAGC	58	520
GAPDH	NM_008084	ACCACAGTCCATGCCATCAC	TCCACCACCCTGTTGCTGTA	58	452
**Real-time PCR**					
ANG1	NM_009640	GCATTCTTCGCTGCCATTCT	TCTCCCTCCGTTTTCTGGATT		
ANG2	NM_007426	CCAACGCCTTAACCCATCTC	ACCCCGAGTCTGTGGATTGAC		
VEGFa	NM_009505	TTGTGTTGGGAGGAGGATGTC	GAAGCCTTTCATCCCATTGTCT		
PLGH	NM_08827	TGGCTGCTGTGGTGATGAA	TGCATAGTGATGTTGGCTGTCTT		
PDGFa	NM_011057	TTTGGAGACTTGGGCTTGGA	AACGGACCCCCAGATCAGA		
GCSF	NM_009971	GCAGGCTCTATCGGGTATTTCC	AGTTGGCAACATCCAGCTGAA		
VCAM1	NM_0011693	TGCGAGTCACCATTGTTCTCAT	ACCCCTCCGTCCTCACCTT		
bFGF	NM_008006	TGGTATGTGGCACTGAAACGA	TCCAGGTCCCGTTTTGGAT		
VEGFR2	NM_010612	ACTGCAGTGATTGCCATGTTCT	TCATTGGCCCGCTTAACG		
Tie2	NM_013690	CTTCATGTACAACGGCCATTTC	GTGGGTGGCTTGCTTGGT		
GAPDH	NM_008084	CCAGCCTCGTCCCGTAGA	CGCCCAATACGGCCAAA		

AT, Anneal Temperature.

Supplementary Table 2: Antibody application

Protein	Assay	Cat. No.	Company	Host	Dilution	Incubation Time
CAR	IF	AF2654	R&D Systems	goat	1:100	O/N, 4°C
T1α	IF	sc23564	Santa Cruz	goat	1:200	O/N, 4°C
AQP5	IF	AB15858	Millipore	rabbit	1:200	O/N, 4°C
CAR	IHC	AF2654	R&D Systems	goat	1:100	O/N, 4°C
Oct-4	IHC	sc5279	Santa Cruz	mouse	1:100	O/N, 4°C
phospho-Src	IHC	ab79308	Abcam	rabbit	1:100	O/N, 4°C
phospho-β-catenin	IHC	ab53050	Abcam	rabbit	1:100	O/N, 4°C
c-myc	IHC	ab32072	Abcam	rabbit	1:500	O/N, 4°C
cycline D1	IHC	ab134175	Abcam	rabbit	1:200	O/N, 4°C
TTF1	IHC	M3575	Dako	mouse	1:100	O/N, 4°C
NAPSA	IHC	NB110-68133H	Novus Biologicals	mouse	1:500	O/N, 4°C
CK7	IHC	ab9021	Abcam	mouse	1:1000	O/N, 4°C
CK-HMW	IHC	ab76714	Abcam	mouse	1:50	O/N, 4°C
CD31	IHC	ab28364	Abcam	rabbit	1:100	O/N, 4°C
CD105	IHC	ab107595	Abcam	rabbit	1:50	O/N, 4°C
vWF	IHC	ab9378	Abcam	rabbit	1:100	O/N, 4°C
Oct-4	WB	sc5279	Santa Cruz	mouse	1:200	O/N, 4°C
Survivin	WB	ab182132	Abcam	rabbit	1:1000	O/N, 4°C
cleaved caspase-3	WB	9664	Cell signaling	rabbit	1:1000	O/N, 4°C
cleaved caspase-9	WB	9509	Cell signaling	rabbit	1:1000	O/N, 4°C
Tie2	WB	sc9026	Santa Cruz	rabbit	1:200	O/N, 4°C
phospho-Tie2	WB	ABS219	Millipore	rabbit	1:2000	O/N, 4°C
ANG1	WB	sc6320	Santa Cruz	goat	1:200	O/N, 4°C
ANG2	WB	2948	Cell signaling	rabbit	1:1500	O/N, 4°C
Grb2	WB	ab32037	Abcam	rabbit	1:1000	O/N, 4°C
ERK	WB	sc93	Santa Cruz	rabbit	1:500	O/N, 4°C
phosphor-ERK	WB	sc7383	Santa Cruz	mouse	1:500	O/N, 4°C
GAPDH	WB	ab181602	Abcam	rabbit	1:1000	O/N, 4°C
CAR	FC	AF2654	R&D Systems	goat	1:100	1 hr, 4°C
CD31-APC	FC	102509	BioLegend		1:100	1 hr, 4°C
CD133-APC	FC	141208	BioLegend		1:100	1 hr, 4°C
Control IgG	IF	012-000-003	Jackson ImmunoResearch	rat	1:200	O/N, 4°C
Control IgG	IF	005-000-003	Jackson ImmunoResearch	goat	1:200	O/N, 4°C
Control IgG	IF	011-000-003	Jackson ImmunoResearch	rabbit	1:200	O/N, 4°C
Control IgG	FC	400511	BioLegend		1:100	1 hr, 4°C
AlexaFlour®488-anti-goat IgG	IF	705-545-003	Jackson ImmunoResearch	donkey	1:200	1 hr, RT
Cy™3-anti-goat IgG	IF	705-165-147	Jackson ImmunoResearch	donkey	1:500	1 hr, RT
Cy™3-anti-mouse IgG	IF	115-165-003	Jackson ImmunoResearch	goat	1:500	1 hr, RT
Cy™3-anti-rabbit IgI	IF	711-165-152	Jackson ImmunoResearch	donkey	1:500	1 hr, RT
AlexaFluor®488-anti-rabbit IgG	IF	111-095-003	Jackson ImmunoResearch	goat	1:500	1 hr, RT

IF, Immunofluorescence; IHC, Immunohistochemistry; WB, Western blot; FC, Flow cytometry; O/N, Overnight; RT, Room temperature.
